# First person – Pavitra Prakash

**DOI:** 10.1242/dmm.049678

**Published:** 2022-06-28

## Abstract

First Person is a series of interviews with the first authors of a selection of papers published in Disease Models & Mechanisms, helping early-career researchers promote themselves alongside their papers. Pavitra Prakash is first author on ‘ Hsp40 overexpression in pacemaker neurons delays circadian dysfunction in a *Drosophila* model of Huntington's disease’, published in DMM. Pavitra is a PhD student in the lab of Dr Sheeba Vasu at Jawaharlal Nehru Centre for Advanced Scientific Research, Bangalore, India, investigating the bi-directional relationship between circadian health and neurodegenerative diseases.


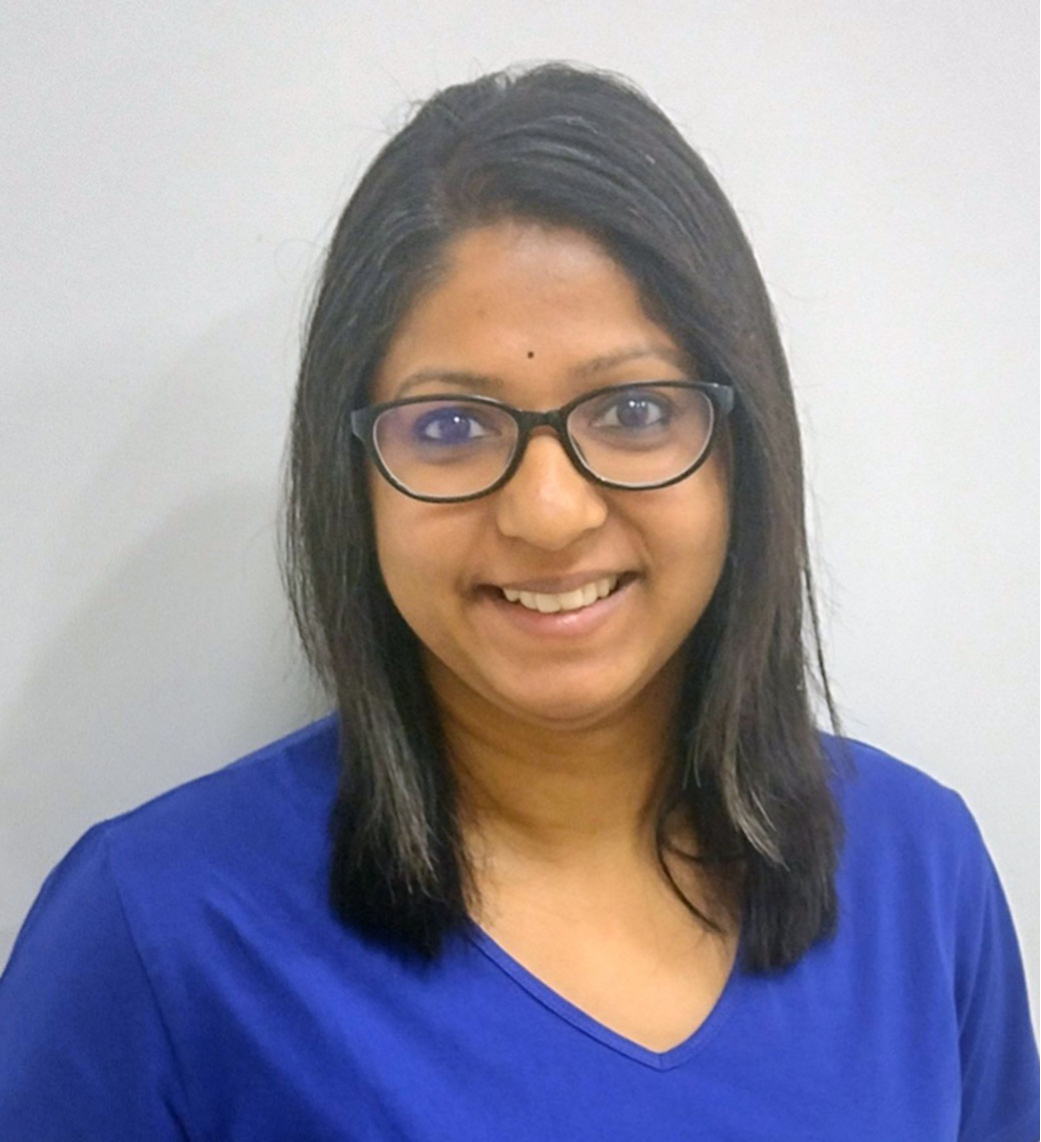


**Pavitra Prakash**

**How would you explain the main findings of your paper to non-scientific family and friends?**

Nearly all living organisms have internal clocks that help time their behaviour and physiology, generating biological rhythms that are integral to their health. Rhythms recurring once every ∼24 h are called circadian rhythms, like the sleep/wake or locomotor activity/rest cycles; these are disrupted in various disease conditions, severely affecting the life quality of patients and caregivers. One such debilitating disease is Huntington's disease (HD), a genetic disease due to a mutant huntingtin (HTT) protein, affecting specific brain regions and leading to loss of motor coordination, cognitive defects and psychiatric issues. We looked for cellular components in the well-studied fruit fly, *Drosophila*, that could overcome circadian disturbances in HD. Induction of HD in the essential clock cells of the fly brain led to a breakdown of activity/rest rhythms and loss of central clock proteins in these neurons. We found that increasing the levels of proteins known as Heat shock proteins (Hsps) in the clock cells of these HD flies suppressed the breakdown of rhythms. Hsps, also known as chaperones, are critical protein quality control elements of the cell that protect neurons against stresses. A subgroup of Hsps, Hsp40, was the most potent circadian booster, delaying rhythm breakdown and restoring circadian proteins in the clock neurons. It also reduced the propensity of mutant HTT protein to form aggregates, often considered toxic and a hallmark of HD. Notably, Hsp40 modified mutant HTT's appearance into a compact oval form that looked like a spot in each cell. Given the circadian rescue, the ‘Spot’ HTT might be relatively less toxic. We provide the first evidence of a significant role for the neuroprotective chaperone Hsp40 in circadian rehabilitation.

“Our results […] strengthen the emerging idea of bi-directional crosstalk between the circadian and neurodegenerative axes.”

**What are the potential implications of these results for your field of research?**

Circadian and sleep disturbances occur early and are pre-manifesting in HD. Therefore, treatments targeting Hsps could impact the early stages of HD, delay symptoms and aid in therapy. Our findings on chaperones' role in circadian rehabilitation open up the possibility of exploring the relatively uncharted area of interactions between the Hsps and the molecular clockwork. Given the importance of protein homeostasis and circadian health in brain and protein aggregation diseases, the involvement of molecular chaperones in circadian maintenance has broader therapeutic implications for several other neurodegenerative diseases like Parkinson's disease and Alzheimer's disease. Our results highlighting the close association between neuronal health and circadian function also strengthen the emerging idea of bi-directional crosstalk between the circadian and neurodegenerative axes. Our study also reveals a need to explore and establish chronotherapeutic neuroprotective agents.

**What are the main advantages and drawbacks of the model system you have used as it relates to the disease you are investigating?**

HD is monogenic and, therefore, one of the more straightforward neurodegenerative diseases to model across various systems. The versatility and genetic tools in *Drosophila*, ease of use for genetic screens, and its well-studied and conserved circadian system make the fly system an obvious choice to screen for modifiers of HD-induced circadian arrhythmia. The fly's short lifespan also allowed us to track pathological changes with age, a key feature in the study of progressive neurodegenerative diseases. A significant advantage of targeting a neuronal circuit *in vivo* that controls an organismal behaviour like the activity/rest rhythm is the ability to probe and intervene at various levels of regulation: molecular, cellular, neuronal circuitry and behavioural. Since the levels are functionally associated, better cause–effect inferences, rigorous testing of the modifying treatment and proof-of-concept evaluations can be made.

Our assays were confined to a subset of critical circadian neurons, cellular markers and circadian activity/rest behaviour. Studying other circadian physiological rhythms, health markers and the biochemistry of mutant HTT in the circadian context would help get a broader understanding. There is variability in the extent of circadian disturbance in HD mice models, and chaperone treatments have differential effects on mice HD pathology, making the study of the effect of chaperones on the mammalian clockwork in neurodegeneration a worthwhile endeavour. Also, since the fly system is relatively simple and less redundant, investigating chaperone interventions on HD-induced circadian dysfunction in mammalian systems like rodents and sheep would provide greater clinical traction.

**Figure:**
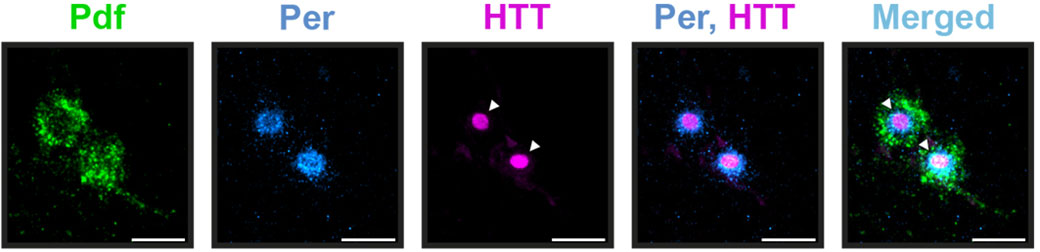
**Mutant Huntingtin put on a spot by Hsp40!** Images show the staining of critical clock neurons, the small ventrolateral neurons in the adult fly brain expressing a circadian neuropeptide Pigment dispersing factor (Pdf; green), the molecular clock protein Period (Per; blue) and mutant Huntingtin (HTT; magenta). Upon overexpressing the chaperone Hsp40 in these clock neurons of flies with Huntington's disease, a new form of mutant HTT appears, the ‘Spot’ form (‘◂’). The Spot HTT overlaps with nuclear Per, is excluded from cytoplasmic Pdf, and is present one in number per cell! Scale bars: 1 μm.

**What has surprised you the most while conducting your research?**

The finding that the co-chaperone Hsp40 was a more potent suppressor of behavioural arrhythmicity than the central chaperone HSP70 was very intriguing. Nevertheless, it was terrific to unveil how the various cellular features also follow a similar pattern of improved rescue with Hsp40. The potential for the nature and function of the Spot form of mutant HTT, which has not been observed in these flies so far, is exciting! A personal lesson while writing this paper has been to be open to telling stories different from what I initially had in mind.

**Describe what you think is the most significant challenge impacting your research at this time and how will this be addressed over the next 10 years?**

These are exciting times for chronotherapy! A unified framework on how neurodegeneration and circadian disruptions feed onto one another needs to be formed and efforts taken to make chronotherapeutics integral to neuromedicine. Also, shifting the debate and research from protein aggregates per se in neurodegeneration to encompass a more inclusive view of the aggregation pathway would be a practical way forward. Given the increasing amount of light pollution, one cannot emphasise and invest enough in chronotherapy, especially on environmental intervention. In the same vein, the current climate of intense competition, social media exposure and screen time demands that, as a society, we prioritise and incentivise better sleep now, before sleep loss further deteriorates healthspan.

**What changes do you think could improve the professional lives of early-career scientists?**

As an early-career researcher, it is crucial to learn to identify and stop projects that are going nowhere or where efforts are disproportionately greater than results. As a PI, it would be desirable to foster a healthy lab environment with a good work–life balance and open communication within the team, have an orientation seminar for new entrees, and encourage active collaborations. The lab team is the life force of the lab, and students often look to their PIs for validation of their work. So, it is paramount to acknowledge their efforts and applaud their accomplishments as significantly (if not more) as admonishments and criticism. A pat on the back goes a long way in making them feel seen, take action and be productive, a win–win scenario!

“A personal lesson […] has been to be open to telling stories different from what I initially had in mind.”

**What's next for you?**

I am looking forward to submitting my PhD thesis and defending it at the earliest. It is a long time coming. I enjoy teaching and love inspiring students to ask questions, think and explore possibilities, fearlessly stand up for themselves and pursue their dreams. My long-term goal is to integrate my background in neuroscience with my emerging interest in behavioural psychology and move into student counselling and mentoring. I dream of cultivating safe spaces to have open conversations on mental health issues and nurturing group therapeutic activities in India's higher education and research institutions.

## References

[DMM049678C1] Prakash, P., Pradhan, A. K. and Sheeba, V. (2022). Hsp40 overexpression in pacemaker neurons delays circadian dysfunction in a *Drosophila* model of Huntington's disease. *Dis. Model. Mech.* 15, dmm049447. 10.1242/dmm.049447 35645202 PMC9254228

